# Improving access to HIV care among people who inject drugs through tele-harm reduction: a qualitative analysis of perceived discrimination and stigma

**DOI:** 10.1186/s12954-024-00961-8

**Published:** 2024-02-23

**Authors:** Carolina Scaramutti, Belén Hervera, Yanexy Rivera, Teresa A. Chueng, David W. Forrest, Edward Suarez, David P. Serota,, Hatoun Alkamli, Katrina Ciraldo, Tyler S. Bartholomew, Hansel E. Tookes

**Affiliations:** 1 Department of Public Health Sciences, University of Miami Miller School of Medicine, Miami, FL USA; 2 2Division of Infectious Diseases, Department of Medicine, University of Miami Miller School of Medicine, Miami, FL USA; 3 Department of Psychiatry and Behavioral Sciences, University of Miami Miller School of Medicine, Miami, FL USA; 4 Department of Family and Community Medicine & Department of Obstetrics, Gynecology and Reproductive Sciences, University of Miami Miller School of Medicine, Miami, FL USA; 5 Division of Health Services Research and Policy, Department of Public Health Sciences, University of Miami Miller School of Medicine, Miami, FL USA

**Keywords:** PWID, HIV, Stigma, Syringe services program, Tele-harm reduction, Harm reduction

## Abstract

**Background:**

Tele-harm reduction (THR) is a telehealth-enhanced, peer-led, harm reduction intervention delivered within a trusted syringe services program (SSP) venue. The primary goal of THR is to facilitate linkage to care and rapid, enduring virologic suppression among people who inject drugs (PWID) with HIV. An SSP in Miami, Florida, developed THR to circumvent pervasive stigma within the traditional healthcare system.

**Methods:**

During intervention development, we conducted in-depth interviews with PWID with HIV (*n* = 25) to identify barriers and facilitators to care via THR. We employed a general inductive approach to transcripts guided by iterative readings of the raw data to derive the concepts, themes, and interpretations of the THR intervention.

**Results:**

Of the 25 PWID interviewed, 15 were in HIV care and adherent to medication; 4 were in HIV care but non-adherent; and 6 were not in care. Themes that emerged from the qualitative analysis included the trust and confidence PWID have with SSP clinicians as opposed to professionals within the traditional healthcare system. Several barriers to treatment were reported among PWID, including perceived and actual discrimination by friends and family, negative internalized behaviors, denial of HIV status, and fear of engaging in care. Facilitators to HIV care included empathy and respect by SSP staff, flexibility of telehealth location, and an overall destigmatizing approach.

**Conclusion:**

PWID identified barriers and facilitators to receipt of HIV care through the THR intervention. Interviews helped inform THR intervention development, centered on PWID in the destigmatizing environment of an SSP.

## Background

People who inject drugs (PWID) are at an increased risk for HIV infection due to both syringe sharing and sexual risk behaviors [1] compounded by competing priorities related to unstable housing and sex work [2–5]. Unfortunately, PWID face substantial barriers to HIV diagnosis, linkage to care, retention in care, and viral suppression [6–8]. Individuals retained in HIV care have a higher likelihood of viral suppression and lower mortality [9]. Stigma is a key barrier to HIV care among PWID [10]. Perceived and experienced discrimination from family and community members can discourage PWID with HIV from seeking medical care [11]. Additionally, internalized stigma can prevent PWID with HIV from accessing services [12]. Both internal and external stigma and experiences with rejection can create fear of disclosing HIV status, further impacting retention in care [5–10, 13, 14].

In cases where PWID have access to antiretroviral therapy (ART), poverty, substance use disorders, mental health disorders, social stigma, and medication side effects can limit adherence. Most patients with HIV report more than one barrier to ART adherence [15]. The HIV care continuum among PWID is disproportionally impacted by social determinants of health, including homelessness, discrimination, and medical distrust [16, 17] with only one in two PWID with HIV virally suppressed in the USA [18, 19].

Effective interventions for reducing HIV transmission among PWID include the provision of drug injection equipment through syringe services programs (SSPs) [20, 21]. SSPs can mediate access to health care services; but, in some cases, implementation has been constrained by law enforcement activities [22]. The evidence is clear: when SSPs were used in combination with other prevention interventions following injection drug-associated HIV outbreaks, there are significant declines in incident HIV infections [23, 24]. However, few studies [25, 26] focus on the relationships between PWID and community members in recovery from addiction, specifically peer workers with lived experience of substance use working at SSPs.

Telehealth has been used to increase access to HIV care for patients who experience challenges along the HIV care continuum, improving their adherence and retention [27]. Residing in the U.S. city with the highest rate of new HIV infections, the IDEA SSP in Miami, Florida, has previously shown how services can be leveraged for linkage to HIV care and substance use treatment [28, 29]. Partnership with the Florida Department of Health has facilitated HIV testing and rapid access to ART [26, 30, 31]. To build on this foundation, we developed Tele-Harm Reduction (THR) to facilitate access to HIV care within the non-stigmatizing environment of a harm reduction setting [32]. The purpose of this qualitative analysis was to identify experiences, perceptions, barriers, and facilitators for engagement of PWID with HIV in care via a THR model. Participants that were interviewed were actively participating in the THR model or had knowledge of the THR pilot intervention and were using SSP services at the time.

## Materials and methods

### Ethics

This study was approved by the Institutional Review Board of University of Miami (IRB# 20190893). All data were de-identified and anonymous.

### Tele-harm reduction intervention

THR has been described in greater detail elsewhere [22]. In brief, tele-harm reduction has two components, meeting PWID where they are physically and emotionally. In component 1, PWID are connected to a physician to initiate ART via telehealth wherever they are (e.g. SSP, mobile unit, encampment). In component 2, a peer with lived experience facilitates ongoing telehealth visits with the physicians and psychologist. Peers may also support adherence by delivering medications stored at the SSP and providing ongoing motivational interviewing.

### Recruitment and participants

Participants were recruited using convenience sampling methods. Inclusion criteria included: (1)18 years of age or older, (2) enrollment at the IDEA Miami SSP, (3) testing reactive via HIV rapid test, (4) reported history of injection drug use, and (5) English- or Spanish-speaking. Pregnant participants were excluded because they may have a unique and non-generalizable experience accessing HIV care. Some interviewees (*n* = 17) were already participating in the THR pilot intervention.

### Semi-structured interviews

After anonymous verbal consent, semi-structured interviews were conducted by a research assistant with previous training in qualitative interviewing and experience working with PWID. Interviews were completed both at the IDEA Miami SSP fixed site and mobile unit in a private setting. Interviews ranged between 15 to 30 min and were audio recorded. Questions were designed to be open-ended, and the interview guide focused on barriers to engagement in HIV care and recommendations for the telehealth intervention, including facilitator characteristics and training. We ascertained knowledge and attitudes regarding HIV treatment for PWID, barriers and facilitators to medication adherence, and long-term retention in HIV care. Table 1 shows the domain and corresponding sample interview questions. All the interviews were transcribed verbatim by an external transcription company.

**Table 1:** Semi-structured interview guide

Domain	
Barriers to engagement in HIV care	What are some of the barriers that [PWID with HIV] have when they want to get into HIV care? What are some of the ways that [PWID with HIV] are treated by others when they want to get into HIV care? What suggestions do you have for making this better?
Perceived benefit of SSP-based care and telehealth	Would [PWID with HIV] feel comfortable coming to a syringe service program like this one to get into HIV care? What are some of the barriers that [PWID with HIV] would have when they want to get into HIV care at a syringe service program? [Telehealth] allows a health care provider to care for a patient when the provider and patient are not physically present with each other by using a computer with a camera and sound so they can see and talk to each other. Would [PWID with HIV] feel comfortable coming to a syringe service program like this one to meet with a case manager or provider using telemedicine?
Recommendations for telehealth test-and-treat	What would be the best location for a syringe services program if it wanted to make it easy for [PWID with HIV] to get into HIV care? What days of the week and times of the day would be best for [PWID with HIV] to get into HIV care at a syringe services program like this one?
Recommendations for facilitator characteristics and training	What should we consider when choosing a staff member at a syringe services program to help [PWID with HIV] to use [telehealth] and to get into HIV care? What are the characteristics of the ideal staff member for this? Would this be different for different types of participants? What types of training or experience should this staff member have?
Knowledge, attitudes, and awareness regarding HIV and treatment for PWID	What are the perceptions/stigma that [PWID with HIV] face? Is there fear that other people will find out about a person’s HIV-positive status if they get into HIV care? What suggestions do you have for overcoming these fears of getting into HIV care for people who inject drugs? What do people who inject drugs think about HIV care and HIV treatment medications?
*For participants with HIV currently in care only*- medication adherence, barriers, and facilitators of long-term adherence and successful strategies used	What are some of the *things that make it easier* for you to take your HIV medications as you should? What are some of the *things that make it harder* for you to take your HIV medications as you should? What things have you done/changes have you made to help you take your HIV medications as you should? What would you need to help make this better?

### Procedure and data analysis

We employed a general inductive approach to understand the barriers and facilitators to care for PWID with HIV at an SSP as presented in the transcribed interviews. A general inductive approach is commonly used in the health and social sciences and allows findings to emerge from the most frequent and dominant codes and themes encountered throughout the analysis [33]. Authors were guided by the interview transcripts to derive the concepts, themes, and interpretations on the objectives.

Following a general inductive approach, CS read all the transcripts while BH, YR, and HA each read two different transcripts. A codebook was created that included code names, definitions, sample quotes, and coding decision rules. The approach entailed data exploration, inductive coding, and thematic analysis. Transcripts were read, re-read, and coded in an iterative fashion. An initial list of themes and subthemes were created. Data saturation was met after the 10th transcript when no new themes or subthemes emerged. Coders then coded one transcript together, discussing coding discrepancies, and adjusting coding decision rules and definitions accordingly. The coding pairs coded transcripts and calculated percent agreement based on consistency of ratings against the lead author (CS). The percentage agreement was calculated to ensure interrater reliability of the codebook. Initial rating of agreement between coding pairs ranged between 90 to 97% on independently coded transcripts, with coding pairs reaching 100% on all final codes.

All final codes were analyzed in Dedoose (Version 8.2.14, Sociocultural Research Consultants, Los Angeles, CA). Specifically, code frequencies were extracted, which allowed for identification of the most highly endorsed codes in the data. Then, the information was extracted on code-cooccurrence to understand the number of times two or more codes appeared together in the same excerpt. Lastly, excerpts from each code were discussed to validate the data and codes and gather sample quotations for our themes and subthemes. To improve the quality of the research and ensure explicit and comprehensive reporting of findings, the consolidated criteria for reporting qualitative research (COREQ) checklist guided the reporting of study methods and results [34].

## Results

There was a total of 25 participants recruited that included 15 participants in HIV care and adherent to medication, 4 in HIV care but non-adherent, and 6 not in care. Age ranged from 23 to 67 years of age (with a mean age of 31 years old); 12 were males, 11 were females. Two participants had missing demographic data (Table 2). Of the 25 participants, 17 (68%) were patients who had received the *THR* pilot intervention and the remaining 8 participants were using SSP services and had knowledge of the THR pilot intervention.

**Table 2:** Participant demographic profile

Characteristic	*N*
Gender	
Male	12
Female	11
Age (years)	
20–30	7
31–40	7
41–50	5
51–60	1
61–70	3
Race/ethnicity	
White (non-Hispanic)	10
White (Hispanic)	9
Black (non-Hispanic)	3
Native American	1

Missing demographic information from 2 participants are not displayed in the profile

**Fig. 1:**
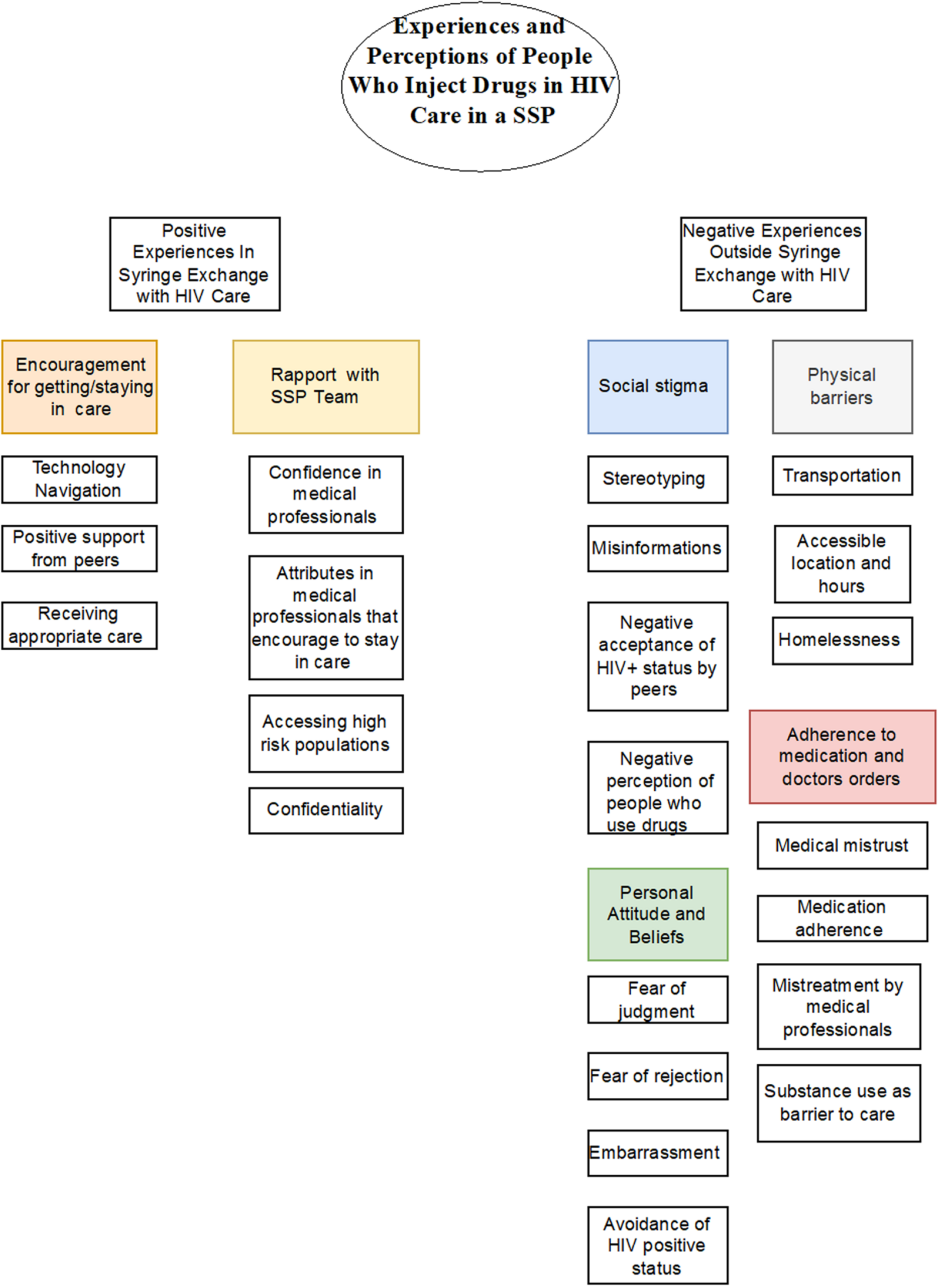
Experiences and perceptions of PWID in HIV care in an SSP

A total of 34 themes were applied 770 times in the 25 transcripts. Themes that appeared most frequently included HIV care, accessing care, confidence in medical doctors, discrimination, medication adherence, negative acceptance by peers, and syringe exchange. Codes that co-occurred the most were: medication adherence and HIV care; syringe exchange and HIV care; negative acceptance by peers and discrimination; syringe exchange and accessible physical location of medical office. Overall, we lumped codes into two themes: barriers to HIV care for PWID and facilitators to engagement, and their corresponding sub-themes (see Fig. 1). Representative quotations from 22 excerpts are provided to ensure substantial evidence of the thematic findings.

### Negative experiences outside syringe exchanges with HIV services

#### Personal attitudes and beliefs: denial of HIV status and internalized stigma

The most common response when asked about barriers to receiving HIV care pertained to participants’ denial or avoidance of their HIV status upon initial diagnosis. Participants frequently reported the fear of being judged due to their HIV status and avoiding certain behaviors or stereotypical locations that would label them as someone who has HIV, such as attending an HIV clinic. Many described waiting until they really had to seek medical treatment to start their care:

“My fear was really bad. I hardly felt comfortably and hardly talked to anyone at all until I ended up having something happen to me that was pretty life threatening or whatever, I was just like, well I gotta start talking. I gotta start doin’ something. I was just so afraid when I found out that I was positive.”

“I didn’t do anything about my HIV until I had to.”

“I didn’t want other people to know. I knew that other people who were using drugs would spread the word that they saw me there and obviously I must have it. That was my biggest fear.”

#### Adherence to medication and doctor’s order: avoidance of medical care in a regular clinical setting

Participants reported difficulty adhering to medication and appointments prior to using the mobile THR pilot due to medical mistrust and mistreatment by professionals. Participants reported mistreatment or humiliation that they encountered at prior medical visits and its effect on their self-esteem and desire for ongoing treatment:

“Everywhere else I've gone, even where I’m staying right now, I was even in a treatment facility, treated me like crap. They don’t help. Even if they have information, they won’t help. I’ve been asking this lady, I signed up for a program and she wants nothing to do with me. It’s a long story, but it had to do with my HIV.”

“The lady that was getting ready to do my blood, she said it out real loud to her coworker, and everybody, even the patient sitting in chairs, make sure you get something, this guy has HIV.”

#### Social stigma

The most in-depth parts of conversation within the semi-structured interviews entailed beliefs regarding HIV and HIV care by SSP participants. Specifically, they described fears of being judged and rejected by the societal community, embarrassment, and feeling alone in their journey with HIV. The fear of judgment was also described as being a hindrance to their retention in HIV care. Participants reported feeling lonely in their care continuum and withdrawn from those who did not know their status. They also reported being fearful that medical professionals would treat them differently because of their HIV status. Below are sample quotations:

“I was worried about people judging me, people knowin’ my business, my personal business getting out, my personal health information getting out on the streets, you know?”

“Well, I have HIV. It’s like you risk outing yourself and putting yourself out there for people to reject you.”

“There’s a fear that everyone will find out about my HIV status and leave.”

Along with personal feelings of judgment and rejection, participants reported that their encounters with those around them were not always positive and cemented their fears of asking for help or strategy of avoiding others. Many participants reported negative encounters with other people, including medical professionals, friends, and family. These encounters took form as stereotyping, incorrect information regarding HIV, negative feelings, and perceptions of persons with HIV and/or associations with drug use. Most commonly, participants reported an over-generalized belief by those they encountered, specifically being labeled as homosexual or a person who uses drugs:

“People look at you—either you’re a homosexual or you’re an IV user—my brother was not. He just slept with the wrong person.”

“The stigma and the way everybody treated my brother before he died. I haven’t told a soul except my doctors because of that.”

“Some people I deal with, even my family, for instance. They were rude to me as far as calling me nasty or sick sometimes. Yeah, people are super cruel. It’s that they’re not educated about it. They’re just stupid, and they say harsh things.”

#### Physical barriers to care

Participants reported external barriers that prevented access to HIV care. Examples included lack of transportation, inaccessible location, and inflexible hours outside of working hours in a traditional clinic. Participants experiencing homelessness had many more difficulties initiating linkage to and maintaining care than those who were stably housed.

“Transportation makes it hard for me to get over and get my meds.”

“I was just gonna say that my only barrier when I’m not regularly on my medication and because I have a dope habit, is that a lot of time this is too far and it’s too hot for me to walk all the way over here. I still don’t have the energy to do it.”

“When I hadn’t got a phone, I couldn’t stay on top of it or contact people to get back into care. Then if you’re on the streets, and just really don't think of it.”

Positive experiences in the Syringe Exchange and Facilitators to HIV Care.

#### SSP as a protective factor staying in care

Participants reported several positive experiences within the SSP-based THR intervention that could facilitate retention in HIV care. All participants had positive remarks regarding the use of THR for HIV care. All participants were accepting of the use of THR for HIV care and utilizing a tele-health modality for services. All participants agreed it would be beneficial for the community to continue to offer this service. SSP participants reported their confidence in HIV screening and counseling services and felt inclined to share this resource with others.

“And probably one of the best resources or means of getting HIV care.”

“I think that it’s basic—you come here to get syringes because you need to use. Then, of course, they help with HIV. Because you use syringes, you—there’s a possible risk that you can get it, or- and it’s all one place, one-stop shop.”

#### Staying in care: encouragement from peers and clinicians at the SSP

Participants were asked if the SSP-based THR intervention was a desired venue for HIV care. All participants reported positive comments regarding their experience with the SSP. Many also cited positive attributes of the personalities of staff that should be emulated among traditional health care provider staff. Although many reported general mistrust with medical professionals, participants considered SSP clinicians trustworthy. Participants described the positivity and welcomeness they felt when visiting the physician at the SSP. Participants reported the unique attributes of staff needed to work with people with HIV and/or PWID. Participants appreciated that the SSP is in an area home to a community placed at high risk, such as PWID and those experiencing homelessness. Participants endorsed the necessity of the SSP and its services for HIV care. Lastly, confidentiality was noted throughout the participant transcripts as being very important when working with patients with HIV:

“The people that are here are very familiar and supportive of people with HIV and other diseases like hepatitis, and things like that. They don’t treat us differently.”

“As far as care, the people here—the caseworkers and everyone—they’re very supportive about it and they try their best to do the most they can to get it to me over and over and over again.”

“Just to let someone know that, if you’re open and honest with me, I’m not going to judge you. I’m still going to give you the best care, something like that to help the person understand that they’re not asking you these questions to judge you or shut you out. They're just trying to help you the best way they can.”

“I think the best thing is to have someone who does a lot more listening than talking, someone that doesn’t appear to be nosy or prying. Because just like in life in general I feel confidentiality is important.”

## Discussion

In this study, we explored the perspectives of PWID with HIV regarding a novel intervention of telehealth-enhanced HIV care delivered through an SSP leveraging the expertise of peer harm reduction counselors (*Tele-Harm Reduction*). Consistent with previous studies, many of these participants reported stigma and discrimination in traditional healthcare settings as the main reasons they discontinued their HIV care or did not seek HIV care. Notable strengths of the *THR* model that facilitated HIV linkage and retention included: (1) nonjudgmental staff, including those with lived experience, who collaborated closely with participants for their HIV and addiction care, and (2) wraparound services in conjunction with telehealth visits that supported comprehensive care at one venue.

Telehealth has been proven to be feasible, cost-effective, and sustainable in SSP settings [35]. Telehealth also reduced the amount of time between an individual’s interest in treatment and getting into treatment and speaking to a provider. This shortening of time provides improved rates in substance use treatment, engagement, and retention [35, 36]. SSPs are often one of the few health-related resources with which PWID regularly engage and therefore fill a unique role in promoting health in a welcoming and non-stigmatizing way [37]. Existing literature reveals that social stigma strongly influences PWID’s healthcare system engagement [38]. Telehealth has been used for direct patient consultation for HIV care, with marginalized populations such as people with HIV in the prison system, and in remote geographical locations [39–41]. The research on the effectiveness of SSPs in reducing injection risk behaviors and HIV transmission dates to 1989 [42]. SSPs that have trust building communication with PWID can reduce and maintain low levels of HIV transmission [43] and further, it has been found that peers reach more diverse networks of PWID [42].

This qualitative assessment of facilitators and barriers to HIV care among PWID frequenting services at an SSP reveal that low-barrier access to compassionate medical care through telehealth could facilitate access to care for a traditionally overlooked cohort. Tookes et al. 2021 [22] reported out of the 35 PWID living with HIV enrolled in the Tele-Harm Reduction intervention, 25 (78%) were virally suppressed at 6 months. A harm reduction framework was employed afterwards to provide on demand access to HIV care among the PWID via remote technology. This telehealth model was integrated to the fixed and mobile site or the location of the patient’s choosing [22]. Centering on PWID, the engagement of peers and linkage to care coordinators in this intervention was of utmost importance. Our study shows that respondent PWID reported their engagement in treatment was influenced by the warm demeanor and non-judgmental ways of staff workers at the IDEA exchange. The majority of those interviewed reported on the stigmatization that they endured in the traditional healthcare setting which ultimately affected their care and treatment, a barrier that could be overcome by the Tele-Harm Reduction intervention.

### Limitations

This study has several limitations. Results may not be generalizable since it was conducted at a single SSP in a single city. Additionally, this secondary analysis of transcripts is limited in scope since the qualitative interviews were done during the implementation of THR and the interview guide was not refined in pursuit of the goals of this analysis in identification of barriers and facilitators. Finally, only one interviewer conducted the interviews, which can introduce interview bias, where the interviewer may subconsciously influence the response of the interviewee. Nonetheless, interviews were conducted within the trusted SSP setting and likely had limited social desirability bias in this context.

### Next steps

This qualitative study elucidated the experience of PWID and their perspective on the importance of destigmatizing provision of HIV care. Due to the promise of the THR intervention, it is currently being tested in a multi-site efficacy trial [44] aiming to transform the way PWID access comprehensive HIV care.

## Data Availability

The data that support the findings of this study are available from the corresponding author upon reasonable request.
